# Comparison of objective quality parameters between CTA and CTP angiographic reconstructions in ischemic stroke patients

**DOI:** 10.1016/j.ejro.2025.100634

**Published:** 2025-01-14

**Authors:** M.M.Q. Robbe, F.M.E. Pinckaers, A.H.H. Dirx, P.H.M. Voorter, W.H. van Zwam, B.A.J.M. Wagemans, A.A. Postma

**Affiliations:** a Department of Radiology and Nuclear Medicine, Maastricht University Medical Center, Maastricht, the Netherlands; b Cardiovascular Research Institute Maastricht (CARIM), University Maastricht, Maastricht, the Netherlands; c Mental Health and Neuroscience Research Institute (MHeNs), University Maastricht, Maastricht, the Netherlands

**Keywords:** Ischemic stroke, Contrast-to-noise ratio, Signal-to-noise ratio, Occlusion detection

## Abstract

**Introduction:**

CT perfusion-angiographic reconstructions (CTP-AR) may be used for occlusion detection in ischemic stroke patients. Objective image quality of CTP-AR needs to be evaluated before implementation as it may affect occlusion detection. In this study, we aimed to assess the objective image quality, by means of contrast to noise ratio (CNR) and signal to noise ratio (SNR), of both CT-angiography and CT perfusion-angiographic reconstructions (CTP-AR).

**Methods:**

Patients with an ischemic stroke between September 2020 up to and including September 2021 who underwent both CT perfusion and CTA at baseline were included. CTP-AR was reconstructed from 1 mm CTP series at the peak arterial enhancement. Per patient, five ipsilateral and five contralateral regions of interest (ROI) were placed. Attenuation and standard deviation per ROI were used to calculate CNR and SNR. Differences in CNR and SNR between CTA and CTP-AR were tested using paired-sample t-tests.

**Results:**

In total, 195/239 patients were included. Both on the ipsilateral and contralateral side, the CNR was significantly higher on CTP-AR compared to CTA (*P* < .001 and *P* < .001, respectively). The SNR measured in the M1 was not significantly different between CTA and CTP-AR (ipsilateral: *P* = .68; contralateral: *P* = .63). The SNR, both on the ipsilateral and contralateral side, was significantly lower on CTP-AR compared to CTA in all parenchyma regions; the caudate nucleus (*P* < .001), lentiform nucleus (*P* < .001), centrum semiovale (*P* < .001), and the parenchyma adjacent to the M1 (*P* < .001).

**Conclusion:**

Image quality measures of CTP-derived angiographic reconstructions indicate higher CNR compared to CTA, but a lower SNR in non-angiographic structures.

## 1 Introduction

In the clinical evaluation of acute ischemic stroke, patients often undergo both computed tomography angiography (CTA) for detecting vascular occlusions and assessing extracranial vascular pathology, and computed tomography perfusion (CTP) for evaluating brain perfusion [1]. Especially due to the expansion of the treatment window for reperfusion therapy, CTP is currently widely available and integrated into the standard clinical work-up [2], [3], [4]. A CTP examination consists of multiple series of CT examination following a contrast bolus over time. Due to advances in CTP technology, i.e. decreasing slice thickness and increasing z-axis coverage, it is currently possible to select a CTP sub-series at the time of maximum arterial inflow and use this for occlusion detection in acute ischemic stroke, which is known as CTP angiographic reconstructions (CTP-AR) [5]. Previous literature has shown that CTP-AR for occlusion detection holds significant potential compared to CTA [5], [6], [7]. CTP-AR may, therefore, be used as a strategy for reducing radiation dose and the volume of contrast medium, as an additional CTA may not be necessary. However, limitations to the clinical application of CTP-AR could include the limited spatial resolution, increased amount of noise, and lower signal-to-noise ratio (SNR) of CTP-AR compared to CTA [5], [8], [9], [10], [11]. In this study, we aimed to assess the objective image quality, by means of contrast to noise ratio (CNR) and signal to noise ratio (SNR), of both CTA and CTP-AR in patients with an ischemic stroke.

## 2 Methods

### 2.1 Study population

This retrospective study enrolled all consecutive emergency room patients diagnosed with ischemic stroke who underwent both CTA and CTP from September 2020 up to and including September 2021. Patients were excluded if aged under 18 years, 1 mm slices of CTP were unavailable, CTP source data was unavailable, severe motion artefacts prevented us from reconstructing the CTP-AR, or if CTA and CTP were not both performed in our centre, as imaging protocols and scan types in primary stroke centres differ.

### 2.2 Imaging protocol

The imaging studies were performed using a 128-multidetector CT system (SOMATOM Flash, Siemens Healthcare, Germany). All patients first underwent CTP followed by CTA.

For the CTP scan, 50 ml of contrast agent (300 mg/ml Iopromide, Ultravist, Bayer, Germany) was injected into the cubital vein at the rate of 7 ml/s followed by a 40 ml saline flush at the same rate. The parameters were as follows: 80 kVp, 100 mAs, and 0.28 s rotation time. The reconstruction kernel was H20f. The z-axis brain coverage was 100 mm, and slice thickness was 1 mm.

For CTA, 45 ml of contrast agent (300 mg/ml Iopromide, Ultravist, Bayer, Germany) was injected at a rate of 5.6 ml/s, followed by a 40 ml/s of saline flush with the same injection rate. After injection, scanning was performed using a computer-assisted bolus tracing program with a trigger threshold of 250 Hounsfield units (HU) in the aortic arch. Scanning started two seconds after the trigger and was performed in caudo-cranial direction. The parameters were as follows: Tube A: 80 kVp/120 mAs, Tube b: Sn 140 kVp/60 mAs, with a rotation time of 0.5 s. Reconstruction kernel was I30f with a weighted average at 0.6. The slice thickness was 1 mm with 0.8 increment.

All CTP images were processed using dedicated post-processing software (Neuro CT perfusion, Syngo.via VB60, Siemens Healthcare, Germany). Using the time-density curve, the 1 mm series at the time of the maximum arterial inflow were selected in order to calculate the CTP-AR.

### 2.3 Objective image quality evaluation

All quantitative measurements were performed using Horos (version 3.3.6). The measurements were performed by a radiology technician in training (A.D) and were visually checked by a neuroradiologist with > 10 years of experience with CTA and CTP (A.P).

**Fig. 1:**
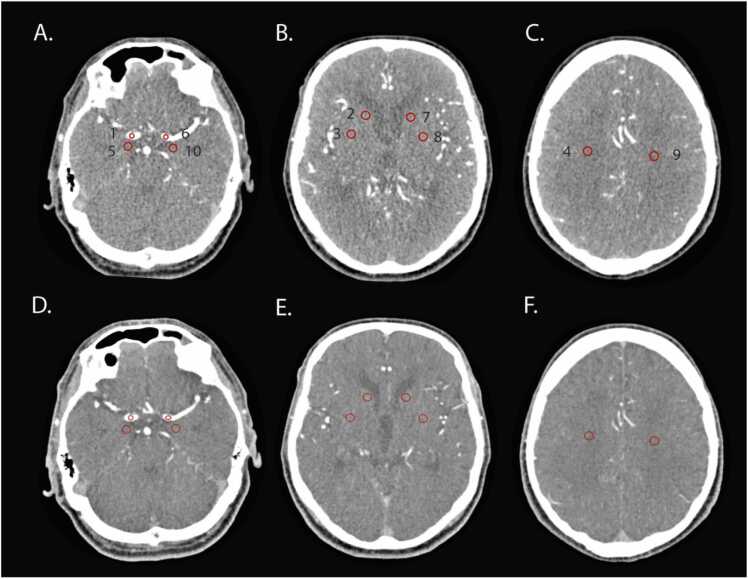
Location of both ipsi- and contralateral measurements on CTP-AR and CTA. Images A-C correspond to the CTP-AR, images D-F correspond to the CTA. The ROI were placed on the following ipsilateral sites: (1) middle cerebral artery (M1) of 10 mm^2^, (2) caudate nucleus of 30 mm^2^, (3) lentiform nucleus of 30 mm^2^, (4) centrum semiovale of 30 mm^2^, (5) the parenchyma adjacent M1 of 30 mm^2^. ROI 6–10 were placed on the same locations but on the contralateral side. Abbreviations: CTA=computed tomography angiography; CTP-AR=computed tomography angiographic reconstructions; ROI=region of interest.

Per patient, five ipsilateral and five contralateral circular regions of interest (ROI) were measured. The ROI were placed in both the CTA and CTP-AR as follows: (1) the middle cerebral artery (M1), 10 mm^2^; (2) the caudate nucleus, 30 mm^2^; (3) the lentiform nucleus, 30 mm^2^; (4) centrum semiovale, 30 mm^2^; (5) the parenchyma immediately dorsal to the M1 ROI, 30 mm^2^ (in order to measure background noise); (Fig. 1). In case of an internal carotid artery terminus (ICA-T) or M1 occlusion, the ROI was placed proximal to the occlusion. The ROI placement was kept as consistent as possible between CTA and CTP-AR. If it was not possible to place a circular ROI in the M1 (as the boundary of the vessel wall would be exceeded), an oval ROI of 10 mm^2^ was placed. Both the mean attenuation (in HU) and the standard deviation (SD) were reported per ROI. The SD was deemed image noise [12].

The following formulas were used to calculate the CNR between the middle cerebral arteries and parenchyma and SNR for all ROIs:

$$\mathrm{CNR}=\;({\mathrm{HU}}_{\mathrm{artery}}–\;{\mathrm{HU}}_{\mathrm{background}})\;/\;\sqrt{{\mathrm{SD}}_{\mathrm{artery}}–\;{\mathrm{SD}}_{\mathrm{background}}};$$

SNR = HU_ROI_ / SD_ROI_ [8], [10], [13].

### 2.4 Statistical analysis

Normality was visually assessed using histograms and Q-Q plots. The CNR and SNR are presented as mean ± SD. Paired-sample t-tests were performed to analyse differences in attenuation, noise, CNR, and SNR between CTA and CTP-AR. Additionally, CNR and SNR were calculated per occlusion location (ICA, ICA-T, M1, M2, M3). P-values < .05 were considered significant. Statistical analyses were performed in R (version 4.3.2).

## 3 Results

**Fig. 2:**
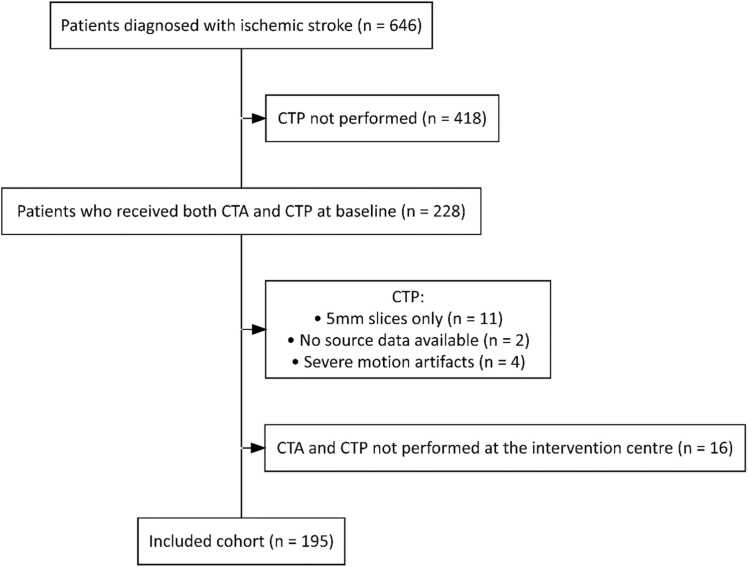
Inclusion flowchart. Abbreviations: CTA=computed tomography angiography; CTP=computed tomography perfusion.

Out of 239 patients eligible, 195 patients were included (Fig. 2). The median age was 77 years (IQR 65–85 years), 99 (51%) patients were male, and the median National Institutes of Health Stroke Scale (NIHSS) was 5 (IQR 3–11) (Table 1). 186 patients directly presented to our intervention centre, while 9 (5%) patients were transferred from a primary stroke centre. In the transferred patients, both CTA and CTP were performed in our hospital.

**Table 1:** Patient characteristics.

	Total (n = 195)
**Patient characteristics**	
Age (years)-median (IQR)	77 (65−85)
Male sex-n(%)	99 (51)
NIHSS score-median(IQR)	5 (3−11)
INR at admission-mean(SD)	1.1 (0.5)
SBP ER (mmHg)-mean(SD)	155 (26)
Intravenous thrombolysis-n(%)	82 (42)
Transfer patient-n(%)	9 (5)
**Medical history-n(%)**	
Previous stroke	44 (23)
Previous myocardial infarction	19 (10)
Previous intracerebral hemorrhage	6 (3)
Atrial fibrillation	27 (14)
Hypertension	78 (40)
Hypercholesterolemia	32 (16)
Peripheral arterial disease	5 (3)
Diabetes mellitus	41 (21)
**Medication and intoxication-n(%)**	
Antihypertensive	98 (51)
Statin	84 (43)
Antiplatelet	83 (43)
DOAC	18 (9)
Vitamin K antagonist	15 (8)
Heparin	6 (3)
Smoking	48 (26)
**Baseline non-contrast CT-median(IQR)**	
ASPECTS	10 (9−10)
**Baseline CT angiography-n(%)**	
Any occlusion	88 (45)
ICA	13 (15)
ICA-T	11 (13)
M1	29 (33)
M2	32 (36)
M3	3 (3)
**Times (mins)-median(IQR)**	
Time from onset to ER	162 (63−594)

Abbreviations: ASPECTS=Alberta Stroke Program Early CT score; DOAC=direct oral anticoagulant; ER=Emergency room; ICA=internal carotid artery; ICA-T = internal carotid artery terminus; INR=International Normalized Ratio; M(segment)=middle cerebral artery; NIHSS=National Institutes of Health Stroke Scale; SBP=systolic blood pressure.

### 3.1 Objective image quality

Both the attenuation (HU) and image noise were significantly higher at all locations on CTP-AR compared to CTA (Table 2).

**Table 2:** Differences in attenuation and noise between CTA and CTP-AR.

	Attenuation (HU)				Noise (HU)			
	CTA (n = 195)	CTP-AR (n = 195)	t-value	P-value	CTA (n = 195)	CTPA (n = 195)	t-value	P-value
M1 ipsilateral*	313 (92)	482 (138)	−22.44	< .001	64 (32)	93 (33)	−11.67	< .001
M1 contralateral	325 (81)	502 (132)	−22.75	< .001	66 (30)	91 (33)	−11.05	< .001
NC ipsilateral	44 (5)	55 (7)	−25.46	< .001	11 (2)	19 (4)	−32.24	< .001
NC contralateral	45 (5)	57 (6)	−26.37	< .001	11 (2)	19 (3)	−30.94	< .001
NL ipsilateral	43 (5)	49 (8)	−11.31	< .001	11 (2)	19 (3)	−30.48	< .001
NL contralateral	43 (5)	49 (8)	−13.35	< .001	11 (2)	19 (3)	−31.07	< .001
Centrum Semiovale ipsilateral	31 (4)	34 (5)	−7.86	< .001	10 (1)	15 (2)	−30.78	< .001
Centrum Semiovale contralateral	31 (4)	34 (5)	−7.99	< .001	10 (2)	15 (3)	−25.71	< .001
Parenchyma adjacent M1 ipsilateral	37 (4)	43 (6)	−11.64	< .001	11 (2)	18 (3)	−30.69	< .001
Parenchyma adjacent M1 contralateral	37 (4)	43 (6)	−12.54	< .001	11 (2)	18 (3)	−30.23	< .001

Note: The first observation represents the CTA and the second observation represents the CTP-AR.

Abbreviations: CTA=computed tomography angiography; CTP-AR=angiographic reconstructions of computed tomography perfusion; HU=Hounsfield units; M1 =segment of middle cerebral artery; NC=caudate nucleus; NL=lentiform nucleus; SD=standard deviation.

* M1 ipsilateral could only be measured in 194 patients, as measurements were precluded in one patient due to the combination of an M1 occlusion and an extracranial carotid stenosis.

In one case, the CNR could not be measured on the ipsilateral side due to an extracranial carotid occlusion in combination with an M1 occlusion. Both on the ipsilateral and contralateral side, the CNR was significantly higher on CTP-AR compared to CTA (*P<*.001 and *P<*.001, respectively; Table 3). The SNR measured in the M1 was not significantly different between CTA and CTP-AR (ipsilateral: *P*=.68; contralateral: *P=*.63). The SNR was significantly lower on CTP-AR compared to CTA in all parenchyma regions; the caudate nucleus (*P*<.001), lentiform nucleus (*P*<.001), centrum semiovale (*P*<.001), and the parenchyma adjacent to the M1 (*P*<.001) (both on the ipsilateral and contralateral side).

**Table 3:** Differences in CNR and SNR between CTA and CTP-AR.

	CTA (n = 195)	CTP-AR (n = 195)	t-value	P-value
**CNR-mean(SD)**				
Ipsilateral*	32.2 (8)	42.4 (12.6)	−13.45	< .001
Contralateral	33.7 (8.1)	44.7 (12.1)	−13.33	< .001
**SNR-mean(SD)**				
M1 ipsilateral*	5.6 (2.3)	5.7 (2.3)	−0.42	.68
M1 contralateral	6.3 (6.6)	6 (2.3)	0.49	.63
NC ipsilateral	4 (0.8)	2.9 (0.7)	18.97	< .001
NC contralateral	4.1 (0.8)	3.1 (0.7)	15.67	< .001
NL ipsilateral	3.8 (0.8)	2.6 (0.6)	21.09	< .001
NL contralateral	4 (0.8)	2.7 (0.6)	19.56	< .001
Centrum Semiovale ipsilateral	3.2 (0.6)	2.2 (0.4)	21.17	< .001
Centrum Semiovale contralateral	3.2 (0.7)	2.3 (0.5)	18.80	< .001
Parenchyma adjacent M1 ipsilateral	3.4 (0.7)	2.4 (0.5)	17.86	< .001
Parenchyma adjacent M1 contralateral	2.4 (0.5)	2.4 (0.5)	15.71	< .001

Note: The first observation represents the CTA and the second observation represents the CTP-AR.

Abbreviations: CNR=contrast-to-noise ratio; CTA=computed tomography angiography; CTP-AR=angiographic reconstructions of computed tomography perfusion; M1 =segment of middle cerebral artery; NC=caudate nucleus; NL=lentiform nucleus; SD=standard deviation; SNR=signal-to-noise ratio.

* M1 ipsilateral could only be measured in 194 patients, due to M1 occlusion and extracranial carotid stenosis.

### 3.2 CNR and SNR per occlusion location

Differences in CNR and SNR between CTA and CTP-AR per occlusion location have been presented in Supplemental Table 1. There was no systematic bias in CNR or SNR of different occlusion locations. CNR was significantly higher in CTP-AR compared to CTA in patients without any occlusion (*P=<*.001), with M1 or M2 occlusions (*P*=.001 and *P=*.001, respectively), while for ICA and ICA-T occlusions the CNR significantly differed only on the contralateral side (*P*=.03 and *P=*.03, respectively). The SNR was significantly higher in CTA compared to CTP-AR in all subgroups, apart from M3 (n = 3).

## 4 Discussion

In this study, we have shown that CTP-AR has a significantly higher CNR between the middle cerebral arteries and background, particularly in patients without an occlusion, compared to CTA. This can be explained by the fact that the CTP is performed at a lower kVp and the CTP-AR represents the CTP series at the point of maximum arterial inflow, which is independent of the time of measurement. As the series at the moment of maximum arterial inflow will demonstrate the best contrast opacification of the vessels, this will result in higher attenuation, thereby explaining the significantly higher CNR for CTP-AR compared to CTA [14]. The SNR of CTP-AR was lower compared to CTA in our present study. Previous literature has described that the SNR is influenced by tube current, slice thickness and patient size [15], [16]. The tube current in our study was higher for CTA compared to CTP. This led to an effective dose of approximately 1,1 mSV for CTA (consisting of one series), and 3,57 mSV for CTP (consisting of 30 series). As a result, considerably less dose per series was used for the CTP-AR compared to the CTA (as the former constitutes the CTP series at the time of maximum arterial inflow), which may explain our findings of a lower SNR in CTP-AR.

Previous literature has also described differences in objective image quality between CTA and CTP-AR [8], [12]. In line with our findings, one study found that attenuation, noise, and CNR were significantly higher in CTP-AR compared to CTA [8]. However, they also found a significantly higher SNR for CTP-AR compared to CTA. As they used a maximum intensity projection of five consecutive time points centered on the peak enhancement phase for reconstruction of CTP-AR, it is possible that the attenuation was relatively enlarged compared to CTA, leading to a significant higher SNR in CTP-AR. Another study found no significant difference between both CNR and SNR on CTA and CTP-AR [12]. They used similar tube currents for both CTA and CTP-AR, while they varied the slice thickness for CTP and CTA (5 mm and 1 mm, respectively) [12]. As slice thickness influences SNR, this likely influenced their findings on SNR in CTA vs. CTP-AR.

The implications of a lower SNR and higher CNR for CTP-AR compared to CTA found in this study depends on whether this influences the diagnostic accuracy of CTP-AR compared to CTA. Previous literature has described a similar diagnostic accuracy for occlusion detection in ischemic stroke between CTP-AR and CTA [5], [6]. This was confirmed in a study with largely the same study cohort as this current study [5]. However, as image noise mostly affects low-contrast detectability, the lower SNR of CTP-AR may have implications in detection of distal thrombi [17], [18]. This previous study, using more or less the same study cohort as the current study, found only a small difference in the number of missed distal M2 and M3 occlusions between CTA and CTP-AR without CTP perfusion maps (5 on CTA versus 7 on CTP-AR, with consensus meeting being the golden standard as not all patients underwent digital subtraction angiography) [6]. The incidence of distal M2 and M3 in this study, however, was relatively low to draw strong conclusions. Therefore it is possible that the SNR of CTP-AR found in this current study, while lower than CTA, remains adequate for detection of (distal) occlusions, but this has yet to be confirmed in future studies using larger sample sizes of distal occlusions.

Strengths of this study include our relatively large cohort and the systematic evaluation of objective image quality of both the ipsi- and contralateral side. Additionally, we kept the slice thickness the same for both CTA and CTP, allowing for a fair comparison of the quality measures between scans. Limitations of this study include the fact that the measurement of M1 ROI may not have been as consistent compared to other ROIs, as a small part of patients had an ICA, ICA-T, or M1 occlusion. As we placed the ROI proximal to the thrombus, it is possible that the placement of the middle cerebral artery ROI slightly differed between the patients. Additionally, these differences in occlusion locations may result in slightly altered contrast opacifications at the M1 region. As the number of proximal occlusions in this study is relatively limited, no systematic bias between occlusion locations was found, and the t-values of both ipsi- and contralateral CNR/SNR are comparable, the expected impact of this limitation seems limited. Moreover, the number of distal vessel occlusions in this study was limited, which is why we were unable to conclude whether the lower CNR/SNR of CTP-AR is of clinical significance in the detection of these distal vessel occlusions. Furthermore, this was a single-centre study, and therefore, generalizability to other scanner types and scan protocols may be limited, though we used standard CTA and CTP protocols.

## 5 Conclusion

Image quality measures of CTP-derived angiographic reconstructions indicate higher CNR compared to CTA, but a lower SNR in non-angiographic structures. Future studies should evaluate whether the lower SNR of CTP-AR results in lower diagnostic accuracy in detection of distal vessel occlusions.

## Ethical approval

Ethical approval for this study was obtained from medical ethics committee of Maastricht University Medical Centre+ , Maastricht, Netherlands (METC-2022–3550).

## Informed consent

The need for individual patient consent has been waived.

## Funding

None.

## Declaration of Competing Interest

WHZ reports speaker fees from Stryker, Cerenovus, Medtronic, Microvention and Nicolab, and consulting fees from Philips (all paid to institution); chaired the advisory boards of WeTrust (Philips) and ANAIS ([Advanced Neurovascular Access in Combination With a Stent Retriever in Patients With Acute Ischemic Stroke]; Anaconda) (all paid to institution); and chaired the advisory board of InExtremis (CHU Montpellier, Montpellier, France) for which no payments were received.

AAP received an institutional grant from Siemens Healthineers and Bayer Healthcare.

Other co-authors have nothing to disclose.
