# Supplementary material for: Comparison of objective quality parameters between CTA and CTP angiographic reconstructions in ischemic stroke patients
Source: Eur J Radiol Open. 2025 Jan 14;14:100634. doi: 10.1016/j.ejro.2025.100634 (PMC11782948; doi:10.1016/j.ejro.2025.100634)
Supplement: Supplementary file 1 — Supplementary material [file mmc1.docx]

| Supplemental Table 1. Results of objective image quality analysis per occlusion location | | | | | | | | | | | | |
| --- | --- | --- | --- | --- | --- | --- | --- | --- | --- | --- | --- | --- |
|  | ICA (n=13) | | ICA-T (n=11) | | M1* (n=29) | | M2 (n=32) | | M3 (n=3) | | None (n=107) | |
|  | t-value | P-value | t-value | P-value | t-value | P-value | t-value | P-value | t-value | P-value | t-value | P-value |
| **CNR** | | | | | | | | | | | | |
| Ipsilateral* | -2.12 | 0.06 | -1.74 | 0.11 | -4.24 | <0.001 | -6.91 | <0.001 | -1.97 | 0.19 | -11.18 | <0.001 |
| Contralateral | -2.53 | 0.03 | -2.68 | 0.03 | -3.62 | <0.001 | -6.55 | <0.001 | -3.10 | 0.09 | -10.52 | <0.001 |
| **SNR** | | | | | | | | | | | | |
| M1 ipsilateral* | 1.21 | 0.25 | 0.82 | 0.43 | -0.12 | 0.9 | 0.12 | 0.91 | -1.65 | 0.24 | -1.20 | 0.23 |
| M1 contralateral | 0.55 | 0.59 | 0.34 | 0.74 | 1.14 | 0.26 | -0.69 | 0.5 | -1.67 | 0.24 | -1.53 | 0.13 |
| NC ipsilateral | 3.99 | <0.001 | 3.43 | 0.01 | 6.99 | <0.001 | 7.68 | <0.001 | 5.28 | 0.03 | 15.03 | <0.001 |
| NC contralateral | 5.16 | <0.001 | 3.95 | <0.001 | 4.93 | <0.001 | 4.58 | <0.001 | 6.59 | 0.02 | 12.86 | <0.001 |
| NL ipsilateral | 7.41 | <0.001 | 5.95 | <0.001 | 8.60 | <0.001 | 8.15 | <0.001 | 5.47 | 0.03 | 14.99 | <0.001 |
| NL contralateral | 4.46 | <0.001 | 3.90 | <0.001 | 11.03 | <0.001 | 8.94 | <0.001 | 1.55 | 0.26 | 13.38 | <0.001 |
| Centrum Semiovale ipsilateral | 7.48 | <0.001 | 4.72 | <0.001 | 8.32 | <0.001 | 8.54 | <0.001 | 1.93 | 0.19 | 15.46 | <0.001 |
| Centrum Semiovale contralateral | 8.23 | <0.001 | 4.13 | <0.001 | 7.20 | <0.001 | 8.15 | <0.001 | 2.72 | 0.11 | 12.91 | <0.001 |
| Parenchyma adjacent M1 ipsilateral | 4.51 | <0.001 | 7.37 | <0.001 | 10.45 | <0.001 | 7.83 | <0.001 | 0.94 | 0.45 | 11.37 | <0.001 |
| Parenchyma adjacent M1 contralateral | 6.40 | <0.001 | 5.47 | <0.001 | 5.37 | <0.001 | 6.97 | <0.001 | 1.40 | 0.3 | 10.82 | <0.001 |
| *Note:*  *M1 ipsilateral could only be measured in 194 patients, due to M1 occlusion and extracranial carotid stenosis.  The first observation represents the CTA and the second observation represents the CTP-AR. | | | | | | | | | | | | |
| Abbreviations: CNR=contrast-to-noise ratio; CTA=computed tomography angiography; CTP-AR=angiographic reconstructions of computed tomography perfusion; M1=segment of middle cerebral artery; NC=caudate nucleus; NL=lentiform nucleus; SD=standard deviation; SNR=signal-to-noise ratio. | | | | | | | | | | | | |
